# pH-Sensitive Co-Adsorption/Release of Doxorubicin and Paclitaxel by Carbon Nanotube, Fullerene, and Graphene Oxide in Combination with *N*-isopropylacrylamide: A Molecular Dynamics Study

**DOI:** 10.3390/biom8040127

**Published:** 2018-10-29

**Authors:** Milad Rezaian, Reza Maleki, Mohammad Dahri Dahroud, Abdolmohammad Alamdari, Milad Alimohammadi

**Affiliations:** 1 Department of Pharmacology, School of Medicine, Shahid Beheshti University of Medical Sciences, 19839-63113 Tehran, Iran; m.rezaian@sbmu.ac.ir; 2 Department of Chemical Engineering, School of Chemical and Petroleum Engineering, Shiraz University, Shiraz 71345, Iran; 3 Student Research Committee, School of Pharmacy, Shiraz University of Medical Sciences, Shiraz 71345, Iran; stud1361866594@sums.ac.ir; 4 Department of Chemical Engineering, Azad University, Kermanshah, Iran; rayanghasr@yahoo.com

**Keywords:** doxorubicin, paclitaxel, *N*-isopropylacrylamide, molecular dynamics, nanotube, release, loading, fullerene, graphene oxide

## Abstract

Nanotechnology based drug delivery systems for cancer therapy have been the topic of interest for many researchers and scientists. In this research, we have studied the pH sensitive co-adsorption and release of doxorubicin (DOX) and paclitaxel (PAX) by carbon nanotube (CNT), fullerene, and graphene oxide (GO) in combination with *N*-isopropylacrylamide (PIN). This simulation study has been performed by use of molecular dynamics. Interaction energies, hydrogen bond, and gyration radius were investigated. Results reveal that, compared with fullerene and GO, CNT is a better carrier for the co-adsorption and co-release of DOX and PAX. It can adsorb the drugs in plasma pH and release it in vicinity of cancerous tissues which have acidic pH. Investigating the number of hydrogen bonds revealed that PIN created many hydrogen bonds with water resulting in high hydrophilicity of PIN, hence making it more stable in the bloodstream while preventing from its accumulation. It is also concluded from this study that CNT and PIN would make a suitable combination for the delivery of DOX and PAX, because PIN makes abundant hydrogen bonds and CNT makes stable interactions with these drugs.

## 1. Introduction

One of the most challenging issues in current medicine, cancer, is receiving more and more global attention in both research and medical practice. Cancer is technically defined as abnormal growth of cells resulting from vicious cell cycle. It has been evident that certain environmental and behavioral factors could predispose individuals to cancer, including radiation exposure, industrial pollutants, smoking, etc. [1]. A backward glance at the extensive scientific work done in the field of cancer reveals that even though much has been known about the etiology, epidemiology, symptoms, diagnosis and treatment of cancer, still a lot remains to be done to uncover the various aspects of this field, especially cancer treatment [2,3,4]. Cancer treatment has nowadays evolved into a multidisciplinary field and various branches of science have contributed to its development, including cellular and molecular biology, genetics, biophysics, biochemistry, and surgery. Common routes of cancer therapy include immunotherapy, chemotherapy, radiotherapy, surgery, etc., and oncologists usually tend to implement combinations of these methods to treat specific malignancies [5,6,7].

Anthracyclines are a class of anticancer medications which induce malignant cell apoptosis by affecting DNA function, inhibiting DNA synthesis, and thereby interfering with cell growth and proliferation. Among the drugs of this class, doxorubicin has been proved to be effective against a wide spectrum of malignancies including lymphoma and breast, thyroid, bladder, stomach, bone, and neural tissue cancers [8,9,10,11,12]. Paclitaxel is another broadly used anticancer medication. It is a member of a class of anticancer drugs called tubulin modulators and has been implemented in the treatment of various malignancies including breast, bladder, lung, esophageal, and ovarian cancers [13]. The drug acts by interfering with microtubules, which are critical intracellular protein structures, and as a result disrupts the normal function and proliferation of the cell. Numerous studies have recently been performed on the effectiveness of doxorubicin/paclitaxel combination therapy. These studies have revealed this combination therapy to be effective for the treatment of breast cancer, hepatocellular carcinoma, lung cancer, brain glioma, esophageal squamous cell carcinoma (SCC), and ovarian cancer [14,15,16].

An important concept in implementing anticancer medications in practical medicine is how the drug is delivered to the body [17]. Various delivery systems have emerged to enhance the specificity of drug delivery to target tissues, among the most important of which are nanotechnology-based drug delivery systems [18,19,20,21,22]. These systems tend to minimize the adverse effects, enhance the drug’s efficacy, prolong its half-life, protect the drug’s molecule, and increase its biocompatibility [23,24]. In such systems, the drug is usually attached to a carbon-based macromolecule called the carrier. The carrier’s proper functions are to slow down the drug’s release and avoid anticancer drugs from affecting normal, noncancerous cells by releasing them in biochemical conditions that are relatively specific to cancerous tissues [18,25,26]. In order to enhance the drug delivery function, a polymer is also attached to the drug–carrier complex. It has been shown that the polymer, by the chemical interactions it makes with both the drug and the carrier, modulates drug adsorption and/or release, prevents undesired carrier or drug aggregations, and increases the solubility and biocompatibility of the drug and the carrier.

Fullerene, nanotubes, and graphene are among the commonly used carriers for anticancer drug delivery. Fullerene is a spherical carbon allotrope. Modified fullerenes can potentially exhibit a lipophilic slow release and significant anticancer activity toward cell culture as proven by C60–paclitaxel conjugate. It has been revealed that even low concentrations of hydrophobic fullerene (20 parts per billion (ppb) level) can induce oxidative damage to cellular membranes. Some studies have shown that encapsulation or micro-encapsulation of fullerene in special carriers such as cyclodextrins and chemical functionalization by amino acid, carboxylic acid, poly hydroxyl group, and amphiphilic polymers can enhance the hydrophilicity and drastically reduce fullerene’s toxicity. The biological efficacy of water-soluble fullerenes has been investigated in vitro, indicating its low toxicity [27,28,29,30,31].

Graphene is a planar sheet carbon allotrope which has been used as nanocarrier in drug delivery systems, thanks to its high surface area and controlled sizes. Possessing a tensile strength of over 100 GPa and a tensile modulus of about 1 TPa, graphene is one of the strongest ever-tested substances [32]. Graphene oxide (GO) is hydrophilic and its surface can be easily modified by a variety of biocompatible polymers including chitosan, polyethylene glycol (PEG), poly(ε-caproplactone), poly-l-lysine (PLL) and polyvinyl alcohol. Owing to its excellent surface area (2600 m^2^ g^−1^), it possesses high drug loading capacity; however, its lack of rigidity results in weak cell penetration [33]. Lateral dimension of GO nanosheets has no impact on their drug-loading capacity, and this fact may impose some limitations in terms of blood–brain transport, renal clearance and biodegradation [34,35,36].

A cylindrical carbon allotrope, carbon nanotube (CNT) has recently drawn the attention of drug delivery researchers [37]. Its surface area and charge, chemical properties, and capability of passing through biological membranes have made CNT a promising candidate for cancer therapy and diagnosis [38,39]. Depending on its diameter and chirality, CNTs exhibit different physical and chemical properties. A well-ordered tubular CNT presents excellent mechanical strength and electrical conductivity. Carbon nanotube diameter is about 1 nm, which is half of a spiral DNA diameter, hence it can pass across membranes and penetrate into cells. Furthermore, its high specific surface area (1300 m^2^ g^−1^) combined with its unique sp^2^-hybridization facilitate its functionalization and lead to a wide range of biomedical applications [40,41,42,43].

Due to the water insolubility of carbon nanostructures, they have a tendency to aggregate in aqueous media through Van der Waals interactions. This aggregation would not be in favor of drug delivery systems because it makes the carrier size bigger and therefore unsuitable for cell penetration, increases the carrier’s toxicity, and avoids its homogenous distribution throughout the blood [44,45,46]. A number of studies have been performed to address this issue. These studies suggest that functionalizing carbon structures with hydrophilic groups could avoid such aggregations and increase the biocompatibility of carriers [47,48]. Hydrophilic polymers can also be used for stabilizing carriers. These polymers are either pH-sensitive or temperature-sensitive. The pH-sensitive polymers, like poly acryl acid, show significantly different adsorption properties in different pH ranges. Temperature-dependent polymers, on the other hand, undergo structural changes as the temperature varies. Temperature-sensitive polymers with lower critical solution temperatures are called LCST’s. These polymers have been the subject of various studies, some of which include: poly acryl amide, poly ethylene glycol methacrylate, and poly vinyl amide [49,50,51,52,53]. 

Molecular dynamics is a newly emerged tool and has been proved to be of great practical value in providing analytic data on pharmaceutical systems, bypassing the monetary and time expenses of conventional experiments [54,55]. Many studies, so far, have investigated anticancer drug delivery systems by carbon nanostructures, but few have been focused on the features of multiple carriers individually and in comparison with each other [34,56,57]. In these molecular dynamics studies, the co-adsorption and co-release of doxorubicin and paclitaxel have been investigated for CNT, graphene, and fullerene in combination with isopropyl acryl amide in both neutral and acidic pH. Furthermore, different parameters of the interactions made among the drugs, carriers, and isopropyl acrylamide have been demonstrated for each carrier and in comparison, with other carriers at the same time.

## 2. Materials and Methods

### 2.1. Molecular Dynamics

Molecular dynamics is based on numerical integration originated from Newton’s motion equations for all of the system particles. Applying Newton’s equations of motion, a set of consecutive atomic positions can be obtained; which can be used in prediction of upcoming moments on the basis of present condition [58]. Molecular dynamics involves the three following stages:

1. Obtaining initial configuration of the particles including characteristics of atoms and the initial velocity as well as physical properties (i.e., mass, size, and type of atom)
2. Calculation of neighbors list made for every atom of the system which contains all atoms within the force range of the targeted atom. This list alters at each step.
3. Calculation of the force applied on each atom based on the configuration, primary conditions and acceleration of each particle as well as the new position and velocity of each particle through integral methods

The continuous nature of relatively real potentials requires breaking of Newton’s motion equations into short time steps (1–10 fs) for integral calculation. Each step involves calculation of the force applied on atoms as well as its merging with the present situations and velocities to obtain the upcoming positions and velocities in the next step. The enacted force of each atom is assumed stable during this time step. As the atoms displace to new positions, a set of forces will be calculated and the process will be repeated. In this content, molecular dynamics simulation offers a path to describe the change of dynamic variables through passing of time [59].

### 2.2. Force Fields

Expressed as a set of mathematical equations, interatomic forces are of great significance in the accuracy and validity of simulated results. Alternation of inter-particle distances will change their interactive energies (potential energy). The potential function is presented in Equation (1); while based on Equation (2), derivation from potential function can lead to force function of each i atom in an N-atom system. These equations are simultaneously solved for short time steps. Besides that, using Equation (3), force can be assigned to the atomic position and time [59,60].

Primary molecular simulation was performed using simple potentials like the hard sphere potential. This model involved particle movement in straight lines with a constant velocity. Relatively elastic collisions happen upon the approach of spheres in a way that their distance becomes equal to the sum of their radii. After collision, new velocities will be calculated based on the principle of conservation of linear motion size. Helpful results are achievable by using the hard sphere model, although it is not ideal for atomic or molecular system simulations. According to the Van der Waals potential, interatomic or intermolecular forces continuously change as their distances vary. However, the hard sphere model considers no force among particles unless they collide with each other. Van der Waals potential is demonstrated in Equation (4), where σ denotes potential well depth and q represents the distance at which the potential becomes zero. Fitting with laboratory data or exact quantum chemistry calculations can be used to determine these parameters. *r* shows the distance between the two atoms, while *V* is their interatomic potential [61].

(1) U=U(r)

(2) $$Fi=-\frac{dv}{dri}\;$$

(3) $$mi\frac{d^{2}ri}{dt^{2}}=Fi,\;i=1,...,N\;$$

(4) $$V_{vdW}=4\varepsilon [{\left(\frac{\sigma }{r}\right)}^{12}-{\left(\frac{\sigma }{r}\right)}^{6}]\;$$

Equations (5) and (6) were used to calculate drug diffusion coefficient. For calculation of drug diffusion coefficient, mean-square displacement (*MSD*) was calculated; coordinates of atoms are also shown as *r* while *t* stands for time. After *MSD* calculation, using Einstein’s relation (Equation (6)), diffusion coefficient can be calculated for the three-dimensional system [52,53,54,55,56,57,58,59,60,61,62].

(5) $$MSD\equiv \;\;\langle {\left[r(t)-r(0)\right]}^{2}\rangle =\frac{1}{t}\sum_{t=t_{0}}^{t}{\left[r(t)-r(0)\right]}^{2}\;$$

(6) $$D=\frac{1}{6}\underset{t\to \infty }{\lim}\frac{MSD}{t}\;$$

Regarding to its popularity, the GROMACS open source software in drug simulation was used. Using Newton’s equations of motion, GROMACS is able to predict the behavior of 100 to 1,000,000 particles. First developed for biomolecules, GROMACS possesses a variety of complex transplant interactions. Regarding its high speed in dealing with non-transplant interactions, GROMACS has also found extensive applications in non-biological systems such as polymers.

### 2.3. System Preparation

Graphene oxide sheets in the dimensions of 20 × 20 Å^2^. Graphene surfaces randomly functionalized the with hydroxyl and epoxide groups. TubeGen Online was employed to make CNT molecules; CNTs surface was covered with carbon dioxide molecules in both protonated and deprotonated modes. Fullerene structure was downloaded from: http://www.nanotube.msu.edu/fullerene/fullerene-isomers.html.

As we used naphthalene structure in the optimized potentials for liquid simulations-all atoms (OPLSaa) force field, the surface charge of carbon atom set to zero. The type of bonds between carbon atoms was defined as phenylalanine, tyrosine, and tryptophan. Angle type was also determined according to the angles of aromatic ring in phenylalanine. Charge and other relevant parameters of this nanostructure functional groups were defined on the basis of similar structures available in OPLSaa force field. Lenard-Jones and Columbian potential models were employed for calculation of non-bond interactions (i.e., electrostatic and Van der Waals). 

The OPLSaa force field was implemented to prepare input structures. To obtain molecule parameters, (changed to script format), all molecules were placed in the box and tip3p water model was utilized as solvent. The energy of all simulation systems was minimized by 50,000 steps. Steepest descent method was employed to omit Van der Waals interactions and create hydrogen bonds between water molecules and other species. Next stage involved gradual rise of system temperature from 0 to 310 K in 100 ps in constant volume using Nose–Hoover algorithm. Furthermore, temperature coupling systems was considered as 0.5 ps. The system was then balanced at constant pressure in 200 ps. The system pressure was balanced by Parrinello–Rahman algorithm. MD simulation was conducted at 37 °C for 50 ns. Cut-off distance was set to 1.2. Particle mesh ewald (pme) was also employed for electrostatic force calculation. Bond lengths were maintained by LINCS (linear constraint solver) algorithm; while SHAKE algorithm was employed to limit the bonds engaged in hydrogen atom which will accelerate the calculations.

## 3. Results and Discussion

### 3.1. Drug–Carrier Interactions

#### 3.1.1. DOX-CNT Interactions

**Figure 1:**
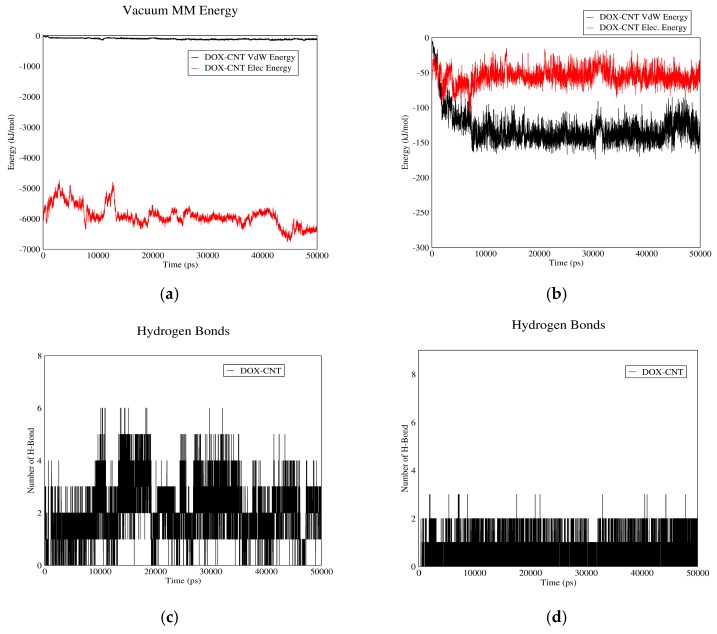
DOX–CNT interaction energies and hydrogen bonds: (**a**) electrostatic and Van der Waals energies of DOX–CNT interaction versus time in neutral pH; (**b**) electrostatic and Van der Waals energies of DOX–CNT interaction versus time in acidic pH; (**c**) the number of hydrogen bonds between DOX and CNT versus time in neutral pH; (**d**) the number of hydrogen bonds between DOX and CNT versus time in acidic pH.

Figure 1 demonstrates DOX–CNT interaction in acidic and neutral pH values. Curves (a) and (b) show Van der Waals and electrostatic interaction energies. Based on these curves, total interaction energies in neutral pH are higher than acidic pH. Therefore, the drug will be adsorbed in neutral pH and released in acidic pH. The plasma pH varies between 7.35 and 7.45, which is nearly neutral. Therefore, the drug will be adsorbed to the carrier in plasma condition and released in vicinity of cancer cells, which have acidic pH due to their higher metabolic rate. Moreover, according to these curves, in neutral pH (adsorption), the interaction between CNT and drug is mainly due to electrostatic forces while in acidic pH (release) Van der Waals interactions make the major contribution to the total drug–CNT interaction energy. The reason could be the presence of carboxylic groups on CNT molecules. In neutral pH, these groups are negatively charged and therefore can better absorb the aromatic and amine groups of the drug. These carboxylic groups will be protonated in acidic pH and their charge will be neutralized.

Figure 1c,d demonstrate the number of H-bonds between drug and CNT versus time in neutral and acidic pH values. As shown in these figures more hydrogen bonds are formed between the drug and CNT in normal pH than acidic pH. The reason is the negative charge of carboxyl groups in neutral pH. This fact would help in adsorption and release of the drug in neutral and acidic pH values, respectively. It would be concluded that CNT can be a good carrier for DOX. It is capable of adsorbing the drug in the bloodstream and releasing it adjacent to cancerous tissues.

#### 3.1.2. DOX–Fullerene Interactions

**Figure 2:**
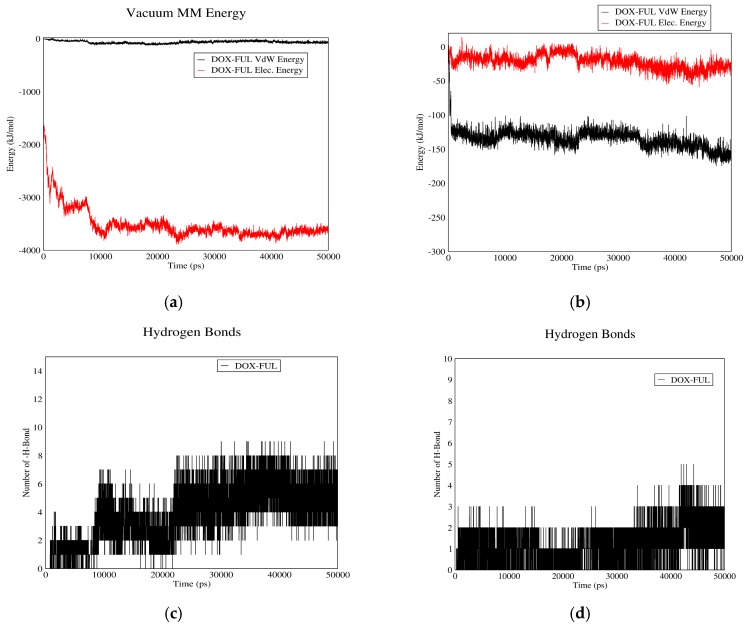
DOX–Fullerene interaction energies and hydrogen bonds: (**a**) electrostatic and Van der Waals energies of DOX–Fullerene interaction versus time in neutral pH; (**b**) electrostatic and Van der Waals energies of DOX–Fullerene interaction versus time in acidic pH; (**c**) the number of hydrogen bonds between DOX and fullerene versus time in neutral pH; (**d**) the number of hydrogen bonds between DOX and fullerene versus time in acidic pH.

Figure 2 demonstrates DOX–fullerene interactions. Based on curves (a) and (b), total interaction energies are significantly higher in neutral pH (plasma pH) than acidic pH (cancerous tissue pH). Therefore, fullerene can adsorb the drug in plasma and release it near cancerous cells. These curves also indicate that Van der Waals energy has no role in DOX–fullerene interaction at neutral pH, in which the total energy is almost equal to the electrostatic energy. The reason could be attributed to the carboxyl groups of fullerene spherical molecules, which by losing their proton, can establish significant electrostatic interactions with the polar functional groups of DOX molecule in neutral pH.

Figure 2c,d show the number of hydrogen bonds between DOX and fullerene as a function of time. As it can be seen, the average number of hydrogen bonds in neutral pH is higher than acidic pH. It is concluded that fullerene could act as a proper carrier for DOX as it adsorbs the drug in blood pH and releases it in acidic pH of cancerous tissue.

#### 3.1.3. DOX–GO Interactions

**Figure 3:**
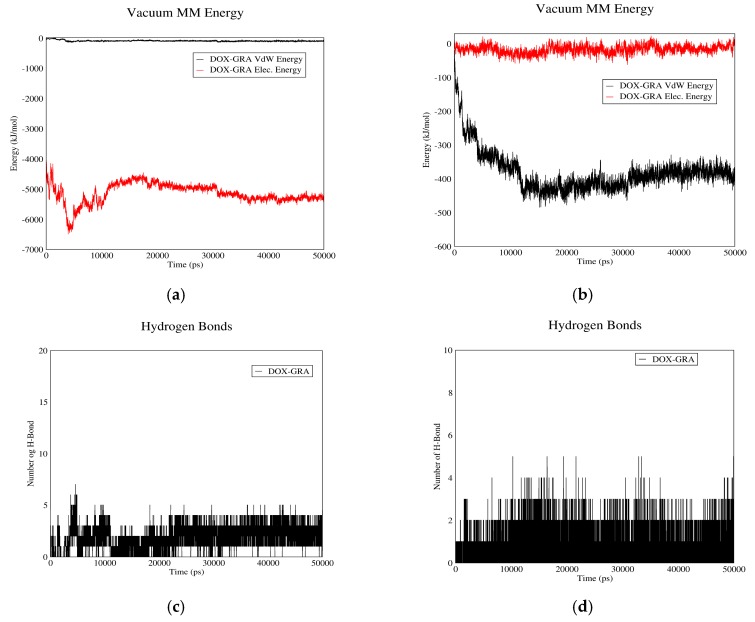
DOX–GO interaction energies and hydrogen bonds: (**a**) electrostatic and Van der Waals energies of DOX–GO interaction versus time in neutral pH; (**b**) electrostatic and Van der Waals energies of DOX–GO interaction versus time in acidic pH; (**c**) the number of hydrogen bonds between DOX and GO versus time in neutral pH; (**d**) the number of hydrogen bonds between DOX and GO versus time in acidic pH.

Figure 3 depicts the interactions between DOX and GO. In curves (a) and (b), Van der Waals and electrostatic energies of drug–carrier interaction are shown in acidic and neutral pH values. As it can be observed, in neutral pH the total interaction energy constitutes almost entirely of electrostatic energy, while this parameter is nearly equal to the Van der Waals energy in acidic pH. The reason is that the protonless carboxylic groups of GO interact with the polar groups of DOX in neutral pH. The mentioned curves also suggest that total interaction energies in neutral pH are significantly higher than acidic conditions. Hence, DOX can be adsorbed on GO in pH values near neutral and released from it in acidic pH.

The number of hydrogen bonds between GO and DOX in neutral and acidic pH values is shown in Figure 3c,d. As these figures suggest, the average number of hydrogen bonds in neutral pH is significantly higher than acidic pH, which would contribute to the drug’s higher tendency to be adsorbed in neutral pH and released in acidic environment. It is concluded that GO can adsorb DOX in the bloodstream and release it near cancerous cells; hence it would be a proper carrier for DOX.

#### 3.1.4. PAX–CNT Interactions

**Figure 4:**
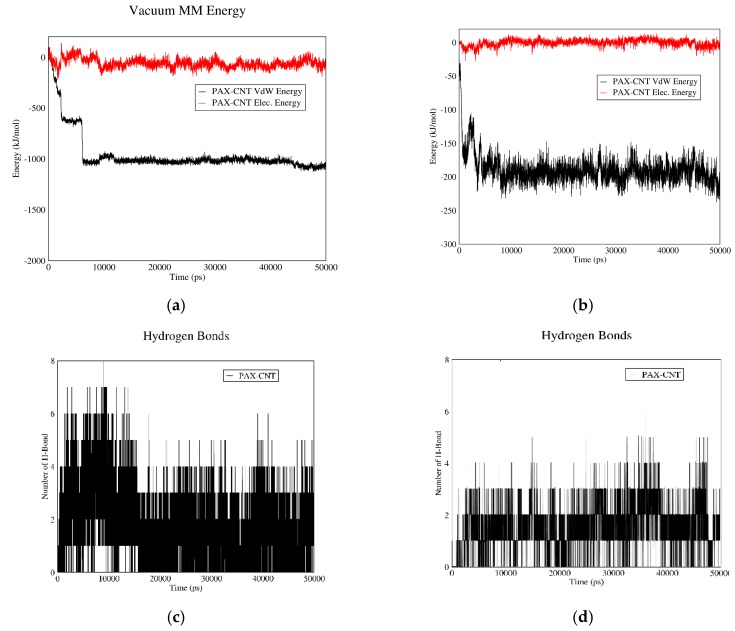
PAX–CNT interaction energies and hydrogen bonds: (**a**) electrostatic and Van der Waals energies of PAX–CNT interaction versus time in neutral pH; (**b**) electrostatic and Van der Waals energies of PAX–CNT interaction versus time in acidic pH; (**c**) the number of hydrogen bonds between PAX and CNT versus time in neutral pH; (**d**) the number of hydrogen bonds between PAX and CNT versus time in acidic pH.

Figure 4 demonstrates PAX–CNT interactions. The interaction energy of PAX and CNT in acidic and neutral pH values versus time are plotted in curves (a) and (b). The total interaction energy in neutral pH is more than this value for acidic pH. Therefore, PAX molecules have the tendency to be adsorbed in neutral pH and released from CNT in acidic pH. These figures also reveal that PAX and CNT have almost no electrostatic interaction in acidic and neutral pH values, and their total interaction energy is mainly due to Van der Waals interactions. The reason is the synthetic nature of PAX molecules; contrary to DOX, they do not possess polar functional groups.

The number of hydrogen bonds between PAX and CNT in both pH values are shown in Figure 4c,d. It is revealed that the average number of hydrogen bonds is more in neutral (adsorption) pH than acidic (releasing) pH. It would be concluded that CNT can serve as a proper carrier for PAX as it adsorbs the drug in the bloodstream and releases it in vicinity of cancerous cells.

#### 3.1.5. PAX–Fullerene Interactions

**Figure 5:**
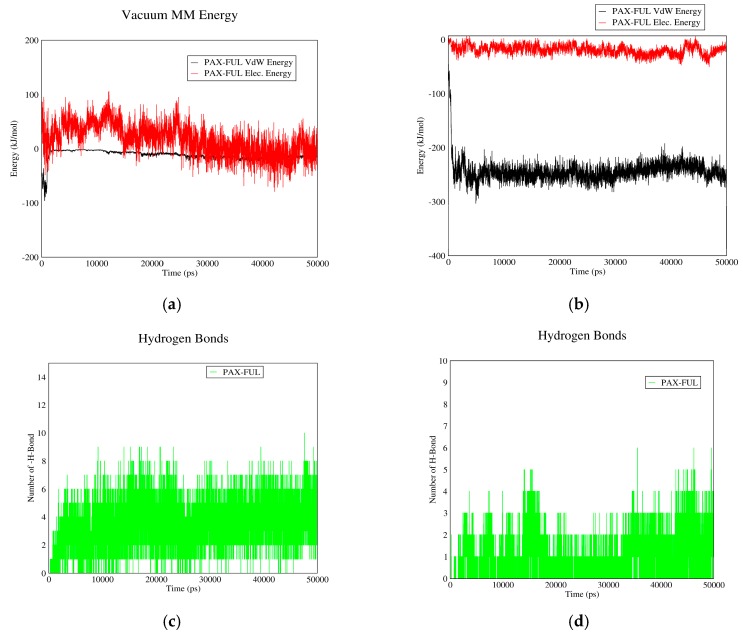
PAX–Fullerene interaction energies and hydrogen bonds: (**a**) electrostatic and Van der Waals energies of PAX–Fullerene interaction versus time in neutral pH; (**b**) electrostatic and Van der Waals energies of PAX–Fullerene interaction versus time in acidic pH; (**c**) the number of hydrogen bonds between PAX and fullerene versus time in neutral pH; (**d**) the number of hydrogen bonds between PAX and fullerene versus time in acidic pH.

Figure 5 shows PAX-fullerene interactions. Based on curves (a) and (b), it can be inferred that PAX and fullerene molecules have almost no electrostatic interactions in neutral or acidic pH. Although relatively small Van der Waals interactions are noted in acidic pH, the values are not that significant to result in the drug’s adsorption or carriage by fullerene molecules. It seems that non-polarity of PAX molecules has resulted in the lack of significant interaction energy between the drug and the carrier.

The number of hydrogen bonds between PAX and fullerene in neutral and acidic pH are depicted in Figure 5c,d. The average number of hydrogen bonds between the two molecules in neutral pH is higher than acidic pH. Although this could be potentially helpful in PAX adsorption in neutral pH and its release from the carrier in acidic pH, it would be concluded, based on Figure 5a,b, that perhaps due to other factors, this has not been effective enough to result in the drug’s higher tendency to be adsorbed/released in acidic/neutral pH. It is concluded that fullerene would not be a proper carrier for PAX.

#### 3.1.6. PAX–GO Interactions

**Figure 6:**
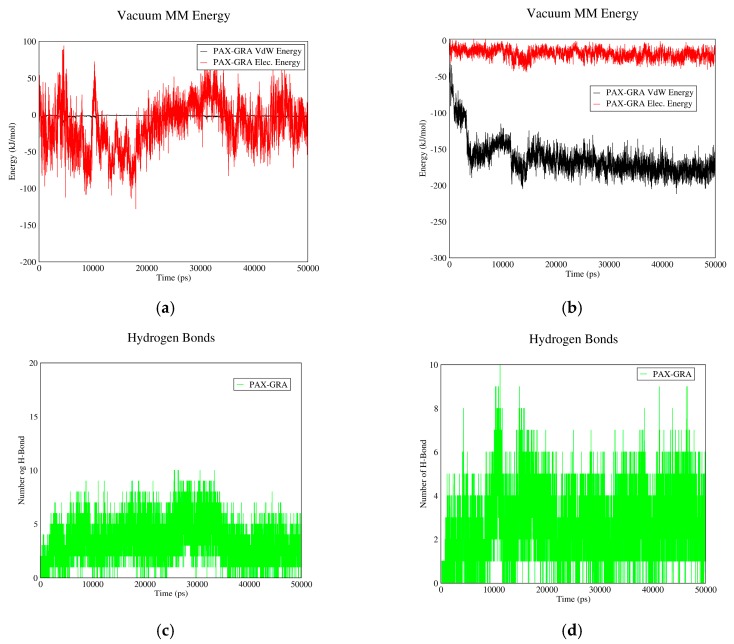
PAX–GO interaction energies and hydrogen bonds: (**a**) electrostatic and Van der Waals energies of PAX–GO interaction versus time in neutral pH; (**b**) electrostatic and Van der Waals energies of PAX–GO interaction versus time in acidic pH; (**c**) the number of hydrogen bonds between PAX and GO versus time in neutral pH; (**d**) the number of hydrogen bonds between PAX and GO versus time in acidic pH.

Figure 6 shows the interactions between PAX and GO. Based on curves (a) and (b), which demonstrate the Van der Waals and electrostatic interactions between PAX and GO, almost no electrostatic interaction exists in acidic or neutral pH as the non-polar PAX molecules cannot have electrostatic interaction with GO. Small Van der Waals energy is detected in acidic pH which is not significant to result in the drug’s adsorption.

Figure 6c,d show the number of hydrogen bonds between PAX and GO. These two figures show no significant difference in the average number of hydrogen bonds for neutral and acidic pH. It is, therefore, concluded that GO would not be a suitable carrier for PAX.

### 3.2. Drug–PIN Interactions

#### 3.2.1. DOX–PIN Interactions

**Figure 7:**
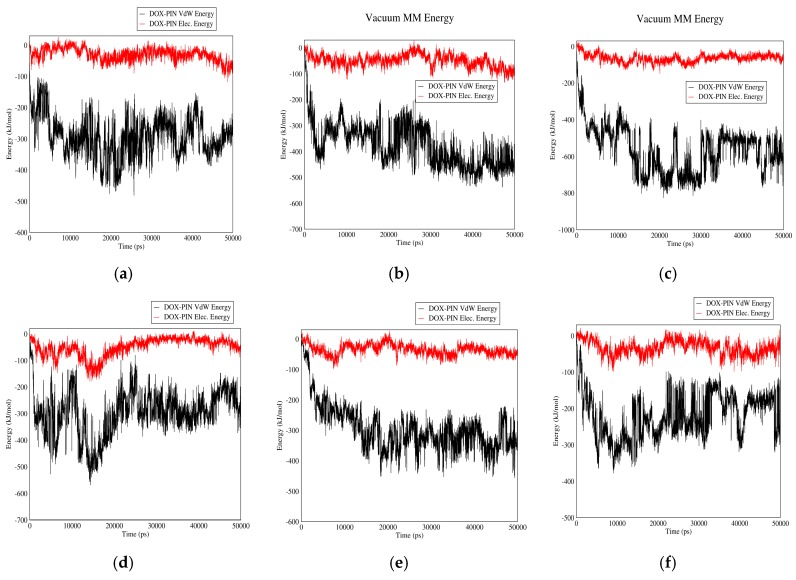
Electrostatic and Van der Waals energies of DOX–PIN interaction versus time in neutral and acidic pH for different carriers: (**a**) CNT in neutral pH; (**b**) fullerene in neutral pH; (**c**) GO in neutral pH; (**d**) CNT in acidic pH; (**e**) fullerene in acidic pH; (**f**) GO in acidic pH.

Figure 7 shows the interactions between DOX and PIN in neutral and acidic environments for different carriers. As it can be observed, electrostatic energy does not play a significant role in DOX-PIN interactions and these molecules interact mainly by Van der Waals energy. These figures also suggest that the interaction between DOX and PIN is affected neither by change of pH nor change of carrier. The absence of significant electrostatic energy could be due to the zero-surface charge of PIN. However, due to Van der Waals interactions and formation of hydrogen bonds between the drug and polymer, the overall solubility of the drug in the blood is enhanced. The polymer also provides good coverage to protect the drug.

#### 3.2.2. PAX–PIN Interactions

**Figure 8:**
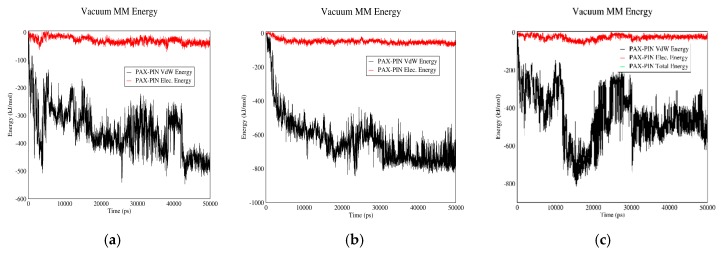

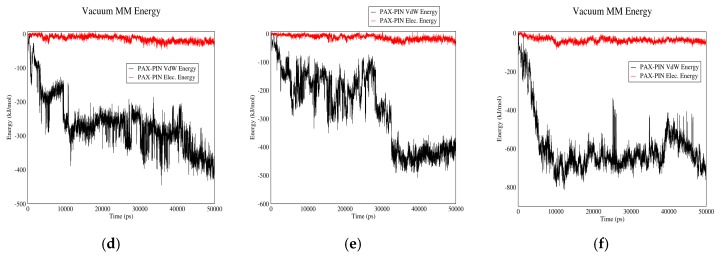
Electrostatic and Van der Waals energies of PAX–PIN interaction versus time in neutral and acidic pH for different carriers: (**a**) CNT in neutral pH; (**b**) Fullerene in neutral pH; (**c**) GO in neutral pH; (**d**) CNT in acidic pH; (**e**) Fullerene in acidic pH; (**f**) GO in acidic pH.

Figure 8 shows electrostatic and Van der Waals interactions between PAX and PIN in acidic and neutral pH values for different carriers. These two molecules have almost similar interactions in both neutral and acidic pH. The interactions are also almost the same for the three carriers. Due to lack of significant electrostatic energy between PAX and PIN, the total interaction energy is nearly equal to the Van der Waals energy. The reason could be lack of surface charge for both PAX and PIN, which leads to lack of significant electrostatic interaction between them.

### 3.3. Comparison of Carriers

#### 3.3.1. DOX–Carrier

**Figure 9:**
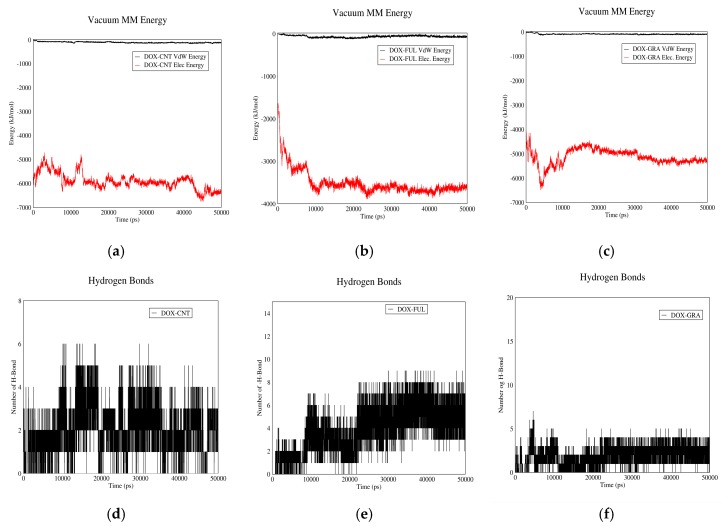
DOX–Carrier interaction energies and hydrogen bonds in neutral pH: (**a**) electrostatic and Van der Waals energies of DOX–CNT interaction versus time in neutral pH; (**b**) electrostatic and Van der Waals energies of DOX–Fullerene interaction versus time in neutral pH; (**c**) electrostatic and Van der Waals energies of DOX–GO interaction versus time in neutral pH; (**d**) the number of hydrogen bonds between DOX and CNT versus time in neutral pH; (**e**) the number of hydrogen bonds between DOX and fullerene versus time in neutral pH; (**f**) the number of hydrogen bonds between DOX and GO versus time in neutral pH.

Figure 9 demonstrates the interactions between DOX and different carriers in neutral pH or adsorption state. Based on curves (a), (b), and (c), all the three carriers can adsorb DOX and their major interaction energy is electrostatic, which arises from the negative charge of the carboxyl groups of carriers and the positive charge of amine groups in the drug molecule. The total interaction energy has the following order CNT > GO > fullerene. In this regard the DOX–carrier bond strength has the following sequence: DOX–CNT, DOX–GO and DOX–fullerene. Figure 9d–f shows the number of hydrogen bonds between DOX and the three carriers in adsorption sate or neutral pH. Based on these figures, the average number of hydrogen bonds between DOX and fullerene is higher than this value for the other two carriers.

**Figure 10:**
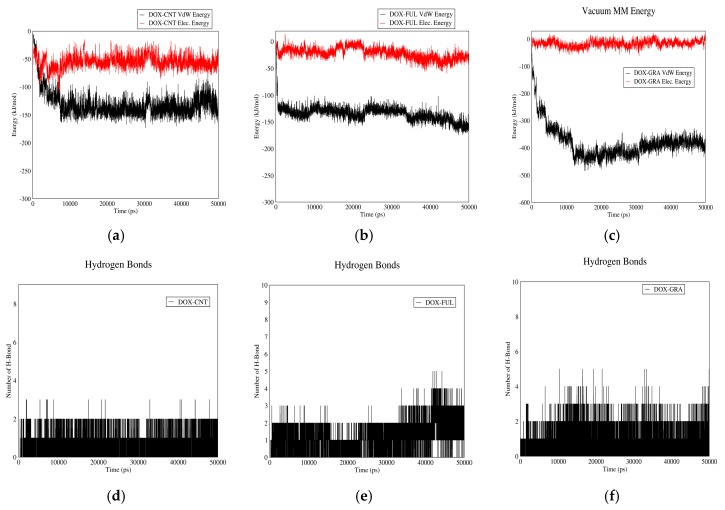
DOX–Carrier interaction energies and hydrogen bonds in acidic pH: (**a**) electrostatic and Van der Waals energies of DOX–CNT interaction versus time in acidic pH; (**b**) electrostatic and Van der Waals energies of DOX–Fullerene interaction versus time in acidic pH; (**c**) electrostatic and Van der Waals energies of DOX–GO interaction versus time in acidic pH; (**d**) the number of hydrogen bonds between DOX and CNT versus time in acidic pH; (**e**) the number of hydrogen bonds between DOX and fullerene versus time in acidic pH; (**f**) the number of hydrogen bonds between DOX and GO versus time in acidic pH.

Figure 10 shows the interactions between DOX and the three carriers in acidic pH (release state). Based on curves (a), (b), and (c), in the acidic pH, the interaction is mainly due to Van der Waals energy, and the total energy has the following descending order: DOX–GO, DOX–CNT and DOX–fullerene. Higher total interaction energy in acidic pH reflects stronger drug-carrier bond in acidic conditions. Therefore, the drug will be released at lower pace in vicinity of cancerous tissues, and its half-life will be increased. So, the half-life of DOX will be more prolonged if it is carried by GO, followed by CNT and fullerene. The number of hydrogen bonds between DOX and the three carriers are shown in Figure 10d–f. Accordingly, in acidic pH, the average number of hydrogen bonds between the drug and carrier is highest in case of GO followed by fullerene and CNT.

#### 3.3.2. PAX–Carrier

**Figure 11:**
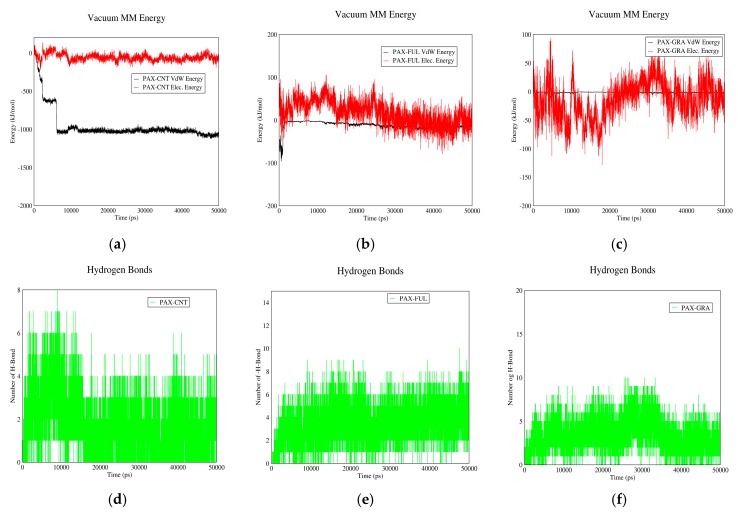
PAX-Carrier interaction energies and hydrogen bonds in neutral pH: (**a**) electrostatic and Van der Waals energies of PAX–CNT interaction versus time in neutral pH; (**b**) electrostatic and Van der Waals energies of PAX–Fullerene interaction versus time in neutral pH; (**c**) electrostatic and Van der Waals energies of PAX–GO interaction versus time in neutral pH; (**d**) the number of hydrogen bonds between PAX and CNT versus time in neutral pH; (**e**) the number of hydrogen bonds between PAX and fullerene versus time in neutral pH; (**f**) the number of hydrogen bonds between PAX and GO versus time in neutral pH.

Figure 11 shows the interactions of PAX with CNT, fullerene and GO in neutral pH values. Based on curves (a), (b), and (c), PAX molecules do not show any significant interaction with fullerene and GO, while they show rather strong interactions with CNT. Therefore, PAX can be adsorbed on CNT in neutral pH. The number of hydrogen bonds between PAX and CNT, fullerene and GO are compared with each other in Figure 11d–f. The average number of hydrogen bonds between CNT and the drug is less than this value for the other two carriers.

**Figure 12:**
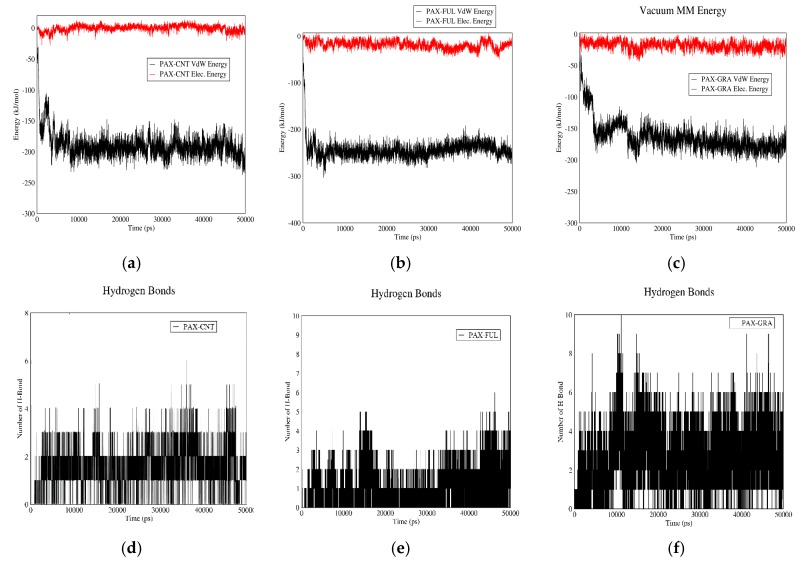
PAX–Carrier interaction energies and hydrogen bonds in acidic pH: (**a**) electrostatic and Van der Waals energies of PAX–CNT interaction versus time in acidic pH; (**b**) electrostatic and Van der Waals energies of PAX–Fullerene interaction versus time in acidic pH; (**c**) electrostatic and Van der Waals energies of PAX–GO interaction versus time in acidic pH; (**d**) the number of hydrogen bonds between PAX and CNT versus time in acidic pH; (**e**) the number of hydrogen bonds between PAX and fullerene versus time in acidic pH; (**f**) the number of hydrogen bonds between PAX and GO versus time in acidic pH.

Figure 12 demonstrates the interactions between PAX and CNT, fullerene and GO in acidic pH. As curves (a), (b), and (c) suggest, Van der Waals interaction makes the major contribution to the total interaction energy at this pH, and these three carriers do not differ significantly in terms of the total interaction energy. Although regarding the previous paragraph, only CNT could serve as a proper carrier for PAX. Figure 12d–f shows the number of hydrogen bonds between PAX and carriers. Based on these figures, it is concluded that the number of hydrogen bonds between PAX and GO is more than this value for the other two carriers.

#### 3.3.3. Radius of Gyration

**Figure 13:**
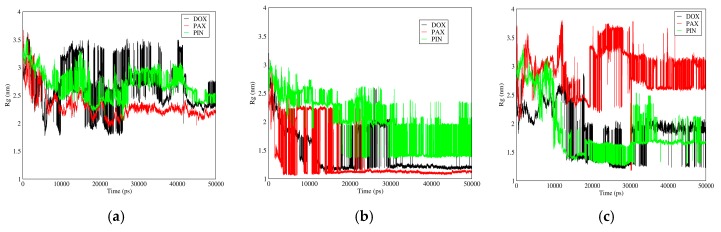

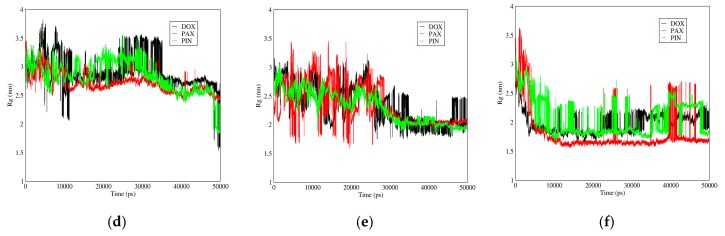
Gyration Radius of DOX, PAX, and PIN versus time in neutral and acidic pH for different carriers: (**a**) CNT in neutral pH; (**b**) Fullerene in neutral pH; (**c**) GO in neutral pH; (**d**) CNT in acidic pH; (**e**) Fullerene in acidic pH; (**f**) GO in acidic pH.

Figure 13 shows gyration radii of DOX, PAX, and PIN for different carriers. In neutral pH, gyration radii of the interacting particles increase by the following order: fullerene, GO, CNT; while in acidic pH, this sequence is GO < fullerene < CNT.

Gyration radius is a factor by which aggregation of molecules (such as polymers) and change of bio-macromolecule size (proteins) can be studied and analyzed. The higher the gyration radius variation is, the stronger the accumulation and polymer-drug interaction will be, and therefore the complex will be more stable.

#### 3.3.4. MSD

**Figure 14:**
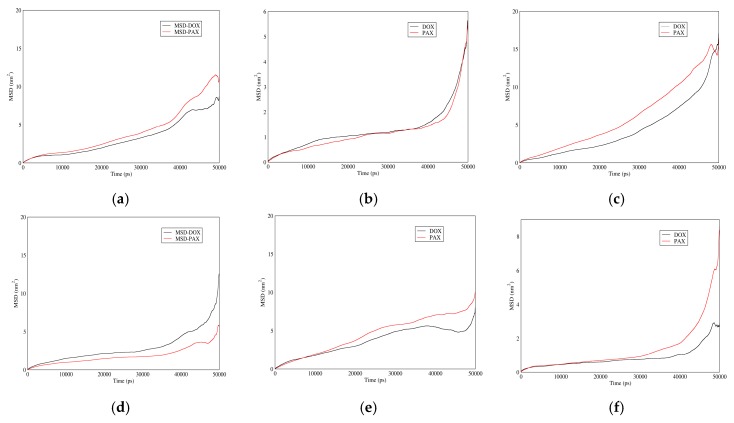
Mean square displacement of DOX and PAX versus time in neutral and acidic pH for different carriers: (**a**) CNT in neutral pH; (**b**) Fullerene in neutral pH; (**c**) GO in neutral pH; (**d**) CNT in acidic pH; (**e**) Fullerene in acidic pH; (**f**) GO in acidic pH.

Figure 14 shows MSD of DOX and PAX for different carriers in acidic and neutral pH values. In neutral pH, MSD of GO is higher than CNT, which itself is higher than fullerene. For acidic pH, MSD of the mentioned drugs is higher for fullerene followed by CNT and GO. It is apparent that the more the MSD is in the adsorption state, the quicker the drug will be absorbed to the carrier. On the other hand, the less the MSD value is in the releasing state, the slower it will be released from the carrier, and therefore the longer will be its half-life. Combining these facts with the above curves, it is concluded that among the three carriers, GO provides the slowest drug release. MSD time-derivative is the molecular diffusion coefficient; therefore, the higher the slope of the curve is, the higher will be the diffusion coefficient.

## 4. Conclusions

DOX and PAX are anticancer drugs which have been used both individually and in combination to treat a wide variety of malignancies. In this molecular dynamics study, the pH-sensitive co-adsorption and co-release of PAX and DOX by CNTs, fullerene, and GO in combination with PIN was investigated.

Results indicated that CNT, fullerene, and GO could be ideal carriers for DOX, among which CNT had the highest binding energy. According to the interaction parameters reported in this study, all of the three carriers had a tendency to adsorb DOX in neutral pH, which is near to the normal pH of plasma, and release it in acidic pH, which, due to a higher metabolic rate, is the pH of cancerous tissues. Because most anticancer drugs, including DOX and PAX, are cytotoxic and can affect normal cells besides cancerous cells, this pH-sensitive drug delivery to cancerous tissues could be of outstanding clinical benefit. For PAX, however, the only carrier which showed such a pH-sensitive response was the CNT.

Furthermore, by analyzing the drugs’ MSD for each carrier, it was observed that among CNT, fullerene, and GO, the latter had the quickest adsorption and slowest release for DOX. The advantage of having a slow release is an increase in the drug’s half-life, and hence an increase in the drug’s injection intervals which would ultimately reduce the total amount of drug delivered to the body.

Moreover, by analyzing the interactions of the drugs and PIN (PIN), it was concluded that this polymer could increase the water solubility of the system and protect the drug’s molecules by covering them. PIN is a temperature-sensitive polymer. Because cancerous tissues are believed to have higher-than-baseline temperatures at least in some of their parts, further researches could be performed to address the efficacy of PIN’s temperature-sensitivity in anticancer drug delivery.

All in all, it was concluded from this study that among CNT, fullerene, and GO, CNT would be a better carrier for the co-transmission of PAX and DOX in combination with PIN, and compared with the classic method of drug injection, CNT-mediated drug delivery could increase the drugs’ half-life by slowing their release, and decrease their side effects by releasing them adjacent to cancerous tissues.

Molecular dynamics simulations provide useful information at molecular level; however, to study interactions in terms of more realistic scales, simulations close to real scales are required. To do so, by means of the data coming from the present simulations, coarse grain simulations can be used, and interaction and bonds resulted from hundreds of molecules of DOX, PAX, PIN and carbon-based materials can be studied within longer spans of time. By increasing the time and length of the system, the PIN–drug and PIN–PIN interactions can be better studied and have a better comparison with laboratory tests. As future works, it is suggested to address the study on pharmaceutical characteristics of CNT–PIN within a greater scale of time and length by means of coarse grain simulations.

## References

[1] Aly H.A. (2012). Cancer therapy and vaccination. J. Immunol. Methods.

[2] Sumanasuriya S., De Bono J. (2018). Treatment of advanced prostate cancer-a review of current therapies and future promise. Cold Spring Harb. Perspect. Med..

[3] Gao C., Liang X., Mo S., Zhang N., Sun D., Dai Z. (2018). Near-infrared cyanine-loaded liposome-like nanocapsules of camptothecin–floxuridine conjugate for enhanced chemophotothermal combination cancer therapy. ACS Appl. Mater. Interfaces.

[4] Oliveri V., Viale M., Caron G., Aiello C., Gangemi R., Vecchio G. (2013). Glycosylated copper(II) ionophores as prodrugs for β-glucosidase activation in targeted cancer therapy. Dalton Trans..

[5] Xu R., Rai A., Chen M., Suwakulsiri W., Greening D.W., Simpson R.J. (2018). Extracellular vesicles in cancer—Implications for future improvements in cancer care. Nat. Rev. Clin. Oncol..

[6] Pastor F. (2016). Aptamers: A new technological platform in cancer immunotherapy. Pharmaceuticals.

[7] Bhattarai P., Hameed S., Dai Z. (2018). Recent advances in anti-angiogenic nanomedicines for cancer therapy. Nanoscale.

[8] Panagiotaki K.N., Sideratou Z., Vlahopoulos S.A., Paravatou-Petsotas M., Zachariadis M., Khoury N., Zoumpourlis V., Tsiourvas D. (2017). A triphenylphosphonium-functionalized mitochondriotropic nanocarrier for efficient co-delivery of doxorubicin and chloroquine and enhanced antineoplastic activity. Pharmaceuticals.

[9] Paleos C.M., Sideratou Z., Theodossiou T.A., Tsiourvas D. (2015). Carboxylated hydroxyethyl starch: A novel polysaccharide for the delivery of doxorubicin. Chem. Biol. Drug Des..

[10] Hameed S., Bhattarai P., Dai Z. (2018). Cerasomes and bicelles: Hybrid bilayered nanostructures with silica-like surface in cancer theranostics. Front. Chem..

[11] Viale M., Giglio V., Monticone M., Maric I., Lentini G., Rocco M., Vecchio G. (2017). New doxorubicin nanocarriers based on cyclodextrins. Investig. New Drugs.

[12] Oliveri V., Bellia F., Viale M., Maric I., Vecchio G. (2017). Linear polymers of β and γ cyclodextrins with a polyglutamic acid backbone as carriers for doxorubicin. Carbohydr. Polym..

[13] Richardson D.L., Sill M.W., Coleman R.L., Sood A.K., Pearl M.L., Kehoe S.M., Carney M.E., Hanjani P., Van Le L., Zhou X.C. (2018). Paclitaxel with and without pazopanib for persistent or recurrent ovarian cancer: A randomized clinical trial. JAMA Oncol..

[14] Tampaki E.C., Tampakis A., Alifieris C.E., Krikelis D., Pazaiti A., Kontos M., Trafalis D.T. (2018). Efficacy and safety of neoadjuvant treatment with bevacizumab, liposomal doxorubicin, cyclophosphamide and paclitaxel combination in locally/regionally advanced, HER_2_-negative, grade III at premenopausal status breast cancer: A phase ii study. Clin. Drug Investig..

[15] Niu L., Zhu F., Li B., Zhao L., Liang H., Yan Y., Tan H. (2018). Folate-conjugated and pH-triggered doxorubicin and paclitaxel co-delivery micellar system for targeted anticancer drug delivery. Mater. Chem. Front..

[16] Rezazadeh M., Akbari V., Amuaghae E., Emami J. (2018). Preparation and characterization of an injectable thermosensitive hydrogel for simultaneous delivery of paclitaxel and doxorubicin. Res. Pharm. Sci..

[17] Catuogno S., Esposito C.L., de Franciscis V. (2016). Aptamer-mediated targeted delivery of therapeutics: An update. Pharmaceuticals.

[18] Lim E.K., Jang E., Lee K., Haam S., Huh Y.M. (2013). Delivery of cancer therapeutics using nanotechnology. Pharmaceutics.

[19] Diaz M.R., Vivas-Mejia P.E. (2013). Nanoparticles as drug delivery systems in cancer medicine: Emphasis on RNAi-containing nanoliposomes. Pharmaceuticals.

[20] Karavolos M., Holban A. (2016). Nanosized drug delivery systems in gastrointestinal targeting: Interactions with microbiota. Pharmaceuticals.

[21] Stockhofe K., Postema J.M., Schieferstein H., Ross T.L. (2014). Radiolabeling of nanoparticles and polymers for pet imaging. Pharmaceuticals.

[22] Zhou J., Shum K.T., Burnett J.C., Rossi J.J. (2013). Nanoparticle-based delivery of RNAi therapeutics: Progress and challenges. Pharmaceuticals.

[23] Debbage P., Thurner G.C. (2010). Nanomedicine faces barriers. Pharmaceuticals.

[24] Ozalp V.C., Eyidogan F., Oktem H.A. (2011). Aptamer-gated nanoparticles for smart drug delivery. Pharmaceuticals.

[25] Johnson R., Sabnis N., McConathy W.J., Lacko A.G. (2013). The potential role of nanotechnology in therapeutic approaches for triple negative breast cancer. Pharmaceutics.

[26] Belleperche M., DeRosa C.M. (2018). pH-control in aptamer-based diagnostics, therapeutics, and analytical applications. Pharmaceuticals.

[27] Kumar M., Sharma G., Kumar R., Singh B., Katare O.P., Raza K. (2018). Lysine-based C_60_-fullerene nanoconjugates for monomethyl fumarate delivery: A novel nanomedicine for brain cancer cells. ACS Biomater. Sci. Eng..

[28] Goodarzi S., Da Ros T., Conde J., Sefat F., Mozafari M. (2017). Fullerene: Biomedical engineers get to revisit an old friend. Mater. Today.

[29] Lapin N.A., Vergara L.A., Mackeyev Y., Newton J.M., Dilliard S.A., Wilson L.J., Curley S.A., Serda R.E. (2017). Biotransport kinetics and intratumoral biodistribution of malonodiserinolamide-derivatized [60]fullerene in a murine model of breast adenocarcinoma. Int. J. Nanomed..

[30] Grebinyk A., Grebinyk S., Prylutska S., Ritter U., Matyshevska O., Dandekar T., Frohme M. (2018). C_60_ fullerene accumulation in human leukemic cells and perspectives of led-mediated photodynamic therapy. Free Radic. Biol. Med..

[31] Thotakura N., Sharma G., Singh B., Kumar V., Raza K. (2017). Aspartic acid derivatized hydroxylated fullerenes as drug delivery vehicles for docetaxel: An explorative study. Artif. Cells Nanomed. Biotechnol..

[32] de Sousa M., Visani de Luna L.A., Fonseca L.C., Giorgio S., Alves O.L. (2018). Folic-acid-functionalized graphene oxide nanocarrier: Synthetic approaches, characterization, drug delivery study, and antitumor screening. ACS Appl. Nano Mater..

[33] Cheng S.J., Chiu H.Y., Kumar P.V., Hsieh K.Y., Yang J.W., Lin Y.R., Shen Y.C., Chen G.Y. (2018). Simultaneous drug delivery and cellular imaging using graphene oxide. Biomater. Sci..

[34] Mahdavi M., Rahmani F., Nouranian S. (2016). Molecular simulation of pH-dependent diffusion, loading, and release of doxorubicin in graphene and graphene oxide drug delivery systems. J. Mater. Chem. B.

[35] Motlagh N.S.H., Parvin P., Refahizadeh M., Bavali A. (2017). Fluorescence properties of doxorubicin coupled carbon nanocarriers. Appl. Opt..

[36] Liu C.-C., Zhao J.-J., Zhang R., Li H., Chen B., Zhang L.-L., Yang H. (2017). Multifunctionalization of graphene and graphene oxide for controlled release and targeted delivery of anticancer drugs. Am. J. Transl. Res..

[37] Khang D.W., Kang S.S., Choi J., Nam T.H. (2018). Carbon Nanotube-Based Anti-Cancer Agent Capable of Suppressing Drug Resistance. U.S. Patent.

[38] Comparetti E.J., Pedrosa V.d.A., Kaneno R. (2017). Carbon nanotube as a tool for fighting cancer. Bioconj. Chem..

[39] Liang L., Shen J.W., Wang Q. (2017). Molecular dynamics study on DNA nanotubes as drug delivery vehicle for anticancer drugs. Colloids Surf. B Biointerfaces.

[40] Geng J., Kim K., Zhang J., Escalada A., Tunuguntla R., Comolli L.R., Allen F.I., Shnyrova A.V., Cho K.R., Munoz D. (2014). Stochastic transport through carbon nanotubes in lipid bilayers and live cell membranes. Nature.

[41] Guven A., Villares G.J., Hilsenbeck S.G., Lewis A., Landua J.D., Dobrolecki L.E., Wilson L.J., Lewis M.T. (2017). Carbon nanotube capsules enhance the in vivo efficacy of cisplatin. Acta Biomater..

[42] Karthik R., Sasikumar R., Chen S.-M., Kumar J.V., Elangovan A., Muthuraj V., Muthukrishnan P., Al-Hemaid F.M., Ali M.A., Elshikh M.S. (2017). A highly sensitive and selective electrochemical determination of non-steroidal prostate anti-cancer drug nilutamide based on *f*-MWCNT in tablet and human blood serum sample. J. Colloid Interface Sci..

[43] Liang L., Chen E.-Y., Shen J.-W., Wang Q. (2016). Molecular modelling of translocation of biomolecules in carbon nanotubes: Method, mechanism and application. Mol. Simul..

[44] Augustine S., Singh J., Srivastava M., Sharma M., Das A., Malhotra B.D. (2017). Recent advances in carbon based nanosystems for cancer theranostics. Biomater. Sci..

[45] Paleos C.M., Tsiourvas D., Sideratou Z., Tziveleka L.A. (2010). Drug delivery using multifunctional dendrimers and hyperbranched polymers. Expert Opin. Drug Deliv..

[46] Kontoyianni C., Sideratou Z., Theodossiou T., Tziveleka L.A., Tsiourvas D., Paleos C.M. (2008). A novel micellar pegylated hyperbranched polyester as a prospective drug delivery system for paclitaxel. Macromol. Biosci..

[47] Bartelmess J., Quinn S., Giordani S. (2015). Carbon nanomaterials: Multi-functional agents for biomedical fluorescence and Raman imaging. Chem. Soc. Rev..

[48] Paleos C.M., Tsiourvas D., Sideratou Z., Tziveleka L. (2008). Multifunctional dendritic drug delivery systems: Design, synthesis, controlled and triggered release. Curr. Top. Med. Chem..

[49] Aihua L., Itaru H., Masaki I., Haoshen Z. (2006). Poly(acrylic acid)-wrapped multi-walled carbon nanotubes composite solubilization in water: Definitive spectroscopic properties. Nanotechnology.

[50] Dong X., Wei C., Liang J., Liu T., Kong D., Lv F. (2017). Thermosensitive hydrogel loaded with chitosan-carbon nanotubes for near infrared light triggered drug delivery. Colloids Surf. B Biointerfaces.

[51] Etika K.C., Jochum F.D., Cox M.A., Schattling P., Theato P., Grunlan J.C. (2010). Nanotube friendly poly*N*-isopropylacrylamide). Macromol. Rapid Commun..

[52] Iglesias D., Bosi S., Melchionna M., Da Ros T., Marchesan S. (2016). The glitter of carbon nanostructures in hybrid/composite hydrogels for medicinal use. Curr. Top. Med. Chem..

[53] Sideratou Z., Agathokleous M., Theodossiou T.A., Tsiourvas D. (2018). Functionalized hyperbranched polyethylenimines as thermosensitive drug delivery nanocarriers with controlled transition temperatures. Biomacromolecules.

[54] Borhani D.W., Shaw D.E. (2012). The future of molecular dynamics simulations in drug discovery. J. Comput.-Aided Mol. Des..

[55] Discher D.E., Ortiz V., Srinivas G., Klein M.L., Kim Y., Christian D., Cai S., Photos P., Ahmed F. (2007). Emerging applications of polymersomes in delivery: From molecular dynamics to shrinkage of tumors. Prog. Polym. Sci..

[56] Cheong D.W., Boon Y.D. (2010). Comparative study of force fields for molecular dynamics simulations of α-glycine crystal growth from solution. Cryst. Growth Des..

[57] Rosano C., Viale M., Cosimelli B., Severi E., Gangemi R., Ciogli A., De Totero D., Spinelli D. (2013). ABCB1 structural models, molecular docking, and synthesis of new oxadiazolothiazin-3-one inhibitors. ACS Med. Chem. Lett..

[58] Zhang L., Peng G., Li J., Liang L., Kong Z., Wang H., Jia L., Wang X., Zhang W., Shen J.-W. (2018). Molecular dynamics study on the configuration and arrangement of doxorubicin in carbon nanotubes. J. Mol. Liq..

[59] Riniker S. (2018). Fixed-charge atomistic force fields for molecular dynamics simulations in the condensed phase: An overview. J. Chem. Inf. Model..

[60] Banerjee P., Roy S., Nair N. (2018). Coarse-grained molecular dynamics force-field for polyacrylamide in infinite dilution derived from iterative Boltzmann inversion and martini force-field. J. Phys. Chem. B.

[61] Huang J., Lemkul J.A., Eastman P.K., MacKerell A.D. (2018). Molecular dynamics simulations using the drude polarizable force field on GOUs with openMM: Implementation, validation, and benchmarks. J. Comput. Chem..

[62] (2005). Molecular dynamics simulation of self- and mutual diffusion coefficients for confined mixtures. J. Chem. Phys..

